# Unbiased Label-Free Quantitative Proteomics of Cells Expressing Amyotrophic Lateral Sclerosis (ALS) Mutations in *CCNF* Reveals Activation of the Apoptosis Pathway: A Workflow to Screen Pathogenic Gene Mutations

**DOI:** 10.3389/fnmol.2021.627740

**Published:** 2021-04-27

**Authors:** Flora Cheng, Alana De Luca, Alison L. Hogan, Stephanie L. Rayner, Jennilee M. Davidson, Maxinne Watchon, Claire H. Stevens, Sonia Sanz Muñoz, Lezanne Ooi, Justin J. Yerbury, Emily K. Don, Jennifer A. Fifita, Maria D. Villalva, Hannah Suddull, Tyler R. Chapman, Thomas J. Hedl, Adam K. Walker, Shu Yang, Marco Morsch, Bingyang Shi, Ian P. Blair, Angela S. Laird, Roger S. Chung, Albert Lee

**Affiliations:** ^1^Centre for Motor Neuron Disease Research, Department of Biomedical Sciences, Faculty of Medicine, Health, and Human Sciences, Macquarie University, North Ryde, NSW, Australia; ^2^Illawarra Health and Medical Research Institute (IHMRI), University of Wollongong, Wollongong, NSW, Australia; ^3^School of Chemistry and Molecular Bioscience and Molecular Horizons, University of Wollongong, Wollongong, NSW, Australia; ^4^Neurodegeneration Pathobiology Laboratory, Queensland Brain Institute, The University of Queensland, St Lucia, QLD, Australia

**Keywords:** proteomics, amyotrophic lateral sclerosis, induced pluripotent stem cell, proteogenomics, cell death, neurodegeneration, proteostasis cell stress and aging, zebrafish

## Abstract

The past decade has seen a rapid acceleration in the discovery of new genetic causes of ALS, with more than 20 putative ALS-causing genes now cited. These genes encode proteins that cover a diverse range of molecular functions, including free radical scavenging (e.g., SOD1), regulation of RNA homeostasis (e.g., TDP-43 and FUS), and protein degradation through the ubiquitin-proteasome system (e.g., ubiquilin-2 and cyclin F) and autophagy (TBK1 and sequestosome-1/p62). It is likely that the various initial triggers of disease (either genetic, environmental and/or gene-environment interaction) must converge upon a common set of molecular pathways that underlie ALS pathogenesis. Given the complexity, it is not surprising that a catalog of molecular pathways and proteostasis dysfunctions have been linked to ALS. One of the challenges in ALS research is determining, at the early stage of discovery, whether a new gene mutation is indeed disease-specific, and if it is linked to signaling pathways that trigger neuronal cell death. We have established a proof-of-concept proteogenomic workflow to assess new gene mutations, using CCNF (cyclin F) as an example, in cell culture models to screen whether potential gene candidates fit the criteria of activating apoptosis. This can provide an informative and time-efficient output that can be extended further for validation in a variety of *in vitro* and *in vivo* models and/or for mechanistic studies. As a proof-of-concept, we expressed cyclin F mutations (K97R, S195R, S509P, R574Q, S621G) in HEK293 cells for label-free quantitative proteomics that bioinformatically predicted activation of the neuronal cell death pathways, which was validated by immunoblot analysis. Proteomic analysis of induced pluripotent stem cells (iPSCs) derived from patient fibroblasts bearing the S621G mutation showed the same activation of these pathways providing compelling evidence for these candidate gene mutations to be strong candidates for further validation and mechanistic studies (such as E3 enzymatic activity assays, protein–protein and protein–substrate studies, and neuronal apoptosis and aberrant branching measurements in zebrafish). Our proteogenomics approach has great utility and provides a relatively high-throughput screening platform to explore candidate gene mutations for their propensity to cause neuronal cell death, which will guide a researcher for further experimental studies.

## Introduction

Proteogenomics is an integrated area of research that blends genomics and proteomics, which was originally described to use proteomics data to improve genome annotations and subsequent protein characterization (Jaffe et al., 2004; Nesvizhskii, 2014). In a typical proteogenomic experiment, high-resolution mass spectrometry is used to identify novel peptides in which the tandem mass spectra are searched against customized databases with predicted protein sequences to provide high-level validation and refinement of gene expression (Nesvizhskii, 2014). Moreover, integrated proteogenomic approaches can be employed to provide increased depth-in-biology to understand better disease mechanisms, perturbations to cellular pathways and dysfunctions in proteostasis arising from genetic variations that are implicated in the pathogenesis of a particular disease (Hedl et al., 2019). While genome-wide association studies (GWAS) have dramatically improved and sped-up the discovery of gene mutations for diseases such as neurodegenerative diseases, various unique challenges arise when trying to determine the downstream biological effects of newly discovered gene variants (Visscher et al., 2017). For example, unlike cancer research where the biology involves uncontrolled proliferation [and therefore cells can be easily immortalized and grown in culture (Mitra et al., 2013) or xenografts (Yada et al., 2018)]; neurodegenerative research is underpinned by finding the causative mechanisms that lead to cell death in specific cell types (e.g., neurons), by which a large population of cells have already perished before a patient even presents with symptoms (Sabatelli et al., 2011; Branch et al., 2016; Woollacott and Rohrer, 2016). This makes sourcing disease-affected neuronal cells from patients extremely challenging, and generating *in vitro* and *in vivo* models to recapitulate the multistep processes for the purposes of biomedical and therapeutic research even more difficult (Al-Chalabi et al., 2014; Morrice et al., 2018; Vucic et al., 2020). Because of the heterogeneity of onset and progression of many neurodegenerative diseases, there have been a number of models published to date reviewed in Van Damme et al. (2017); Morrice et al. (2018) that recapitulate various phenotypic aspects of disease (e.g., memory deficits). However, none are considered the ‘classic’ or ‘benchmark’ models given the numerous and converging signaling pathways that all affect the biology differently.

Amyotrophic lateral sclerosis (ALS) is typically a late-onset neurodegenerative disease characterized by the selective degeneration of upper and lower motor neurons of the cerebral cortex, brainstem, and spinal cord. It is the most common form of adult motor neuron disease (MND) with poor prognosis and limited treatment options. Given the complexity in molecular origins of ALS, it is not surprising that a catalog of molecular pathways has been implicated in the disease. These include disruption in DNA/RNA processing (Vance et al., 2009; Mitchell et al., 2013), oxidative stress (Duan et al., 2010), excitotoxicity (King et al., 2016), protein misfolding and aggregation (Ciryam et al., 2017; Yerbury et al., 2020), protein degradation (ubiquitin-proteasome and autophagy) (Shahheydari et al., 2017), proteostasis dysfunction (McAlary et al., 2019), ER stress (Jaronen et al., 2014; Prell et al., 2019), and neuroinflammation (Olesen et al., 2020). While environmental factors may play a role in the pathogenesis of the disease, the only established cause of ALS is genetic mutations. More than 20 putative ALS-causing genes are now cited and the pace of discovery is continually accelerating (Nguyen et al., 2018). However, one challenge that arises during genetic mutation discovery is determining whether newly discovered genetic variants are indeed causative of disease or are neutral mutations (i.e., mutations that do not cause a biological effect) (Lattante et al., 2020). Current methods use a range of bioinformatic prediction tools to classify whether gene variants are possibly pathogenic (Lattante et al., 2020), but few have used an experimental workflow to screen the activation of molecular pathways that cause apoptosis (Hogan et al., 2017). High-throughput technologies such as proteogenomics can advance our understanding of both primary pathological proteins and associated proteins to gain insights into the biological effects of potential gene mutations (Hedl et al., 2019).

We have previously identified novel missense mutations in *CCNF* in patients with ALS and FTD (Williams et al., 2016). *CCNF* encodes for cyclin F, a 786 amino acid protein that forms part of the multi-protein Skp1-Cul1-F-Box (SCF^*Cyclin F*^) E3 ligase that is responsible for tagging substrates with ubiquitin for degradation by the proteasome. Protein degradation via the ubiquitin-proteasome system (UPS) involves three key steps, activation of ubiquitin by E1s, ubiquitin conjugation by E2s and ligation of protein substrates by E3s. Activation of this sequential process by ATP results in the covalent attachment of ubiquitin to specific lysine residues on target proteins, with specific polyubiquitin linkages generally marking them for degradation by the UPS (Lys48) or autophagy (Lys63) pathways. There are over 500 E3 ligases in the human proteome with each E3 being capable of binding their own specific set of substrates (Nakayama and Nakayama, 2006; Rayner et al., 2019). To date, the known substrates of cyclin F include RRM2 (D’Angiolella et al., 2012), CDC6 (Walter et al., 2016), CP110 (D’Angiolella et al., 2010), NuSAP (Emanuele et al., 2011), E2F (Clijsters et al., 2019), Exo1 (Elia et al., 2015), and SLBP (Dankert et al., 2016), all of which play a role in cell cycle progression and in DNA synthesis and maintaining genome stability (Galper et al., 2017). The cellular role of cyclin F in non-dividing cells such as neurons is presently not known; however, recent studies have revealed protein-protein interactions between cyclin F and ALS-associated proteins TDP-43, VCP and p62 (Yu et al., 2019).

We recently characterized the Ser621Gly (S621G) mutation in cyclin F, which induced apoptosis pathways and caused motor neuron axonopathies and reduced motor response in zebrafish (Hogan et al., 2017). Mechanistically, we also found that the enzymatic activity was aberrantly elevated in the mutant protein which results in the increased Lys-48 ubiquitylation of the proteome (Lee et al., 2017a, b). This suggests that hyperubiquitylation of protein substrates would also concomitantly increase protein trafficking and potentially impair proteasome activity as the cells’ maximal degradation capacity is reached, leading to accumulation of ubiquitylated proteins and apoptosis. However, our studies have indicated that proteasome function remains unchanged and intact in neuronal cells expressing *CCNF* mutations (Williams et al., 2016) which suggests that cyclin F mutations triggers apoptosis through other mechanisms. While numerous other possible cellular mechanisms may link the S621G mutation to neurodegeneration, validating each hypothesis requires hundreds of biochemical assays to be tested. Given that other cyclin F mutations were also discovered and potentially linked to both sporadic and familial ALS and FTD in our previous GWAS study (Williams et al., 2016), we wanted to develop a relatively high-throughput, quick and informative strategy that could screen additional *CCNF* mutations (K97R, S195R, S509P and R574Q) to determine whether i) they were disease-causing and therefore used as model systems, and ii) verified by other biochemical and *in vivo* studies.

A direct method and output for determining whether a genetic mutation is disease-causing could be the determination of whether the apoptosis pathways have been activated in a model system expressing the chosen gene variant. Apoptosis can be activated by a range of exogenous and endogenous stimuli, such as DNA damage, ischemia, and oxidative stress (Payne et al., 1995). It plays an important function in development and in the elimination of damaged cells, which is facilitated by the release of cytochrome c from the mitochondria through the Bcl-2 family of regulatory proteins. Members of the Bcl-2 family of apoptotic molecules are substrates of the Lys-48 ubiquitin degradation process, and the regular turnover of these molecules by the UPS is an important factor in maintaining the balance between pro- and anti-apoptotic members of this family (Chen and Qiu, 2013). These proteins are classified into subfamilies according to their functionalities and their number of Bcl-2 homology (BH) domains. There are three subfamilies: the anti-apoptotic, which includes Bcl-2, Bcl-XL, Bcl-W, and MCL-1, the multi-BH protein effectors such as Bax and Bak and the BH_3_-only proteins, Bad, Bim, Puma, and Bid. These proteins regulate the permeabilization of the mitochondrial outer membrane (MOMP) and therefore apoptosis via a complex network of heterodimeric interactions (Kale et al., 2018).

The direct activation model suggests that the anti-apoptotic members of the Bcl-2 family suppress apoptosis by sequestering the BH_3_-only proteins, while other BH_3_-only pro-apoptotic members compete to release activating pro-apoptotic proteins (Shamas-Din et al., 2013). Another model, the neutralization model (Chen et al., 2005) suggests that the pro-apoptotic activity of Bax and Bak is regulated by the anti-apoptotic proteins and upon activation, the BH_3_-only proteins bind with and neutralize the anti-apoptotic proteins, allowing Bax and Bak to initiate apoptosis setting off a cascade of proteolytic events to activate caspase-3 and caspase-7 (Kalkavan and Green, 2018). Regardless of the exact mechanism, the delicate balance of pro- and anti-apoptotic molecules is essential for maintaining cellular homeostasis, which can be used to verify whether a newly discovered gene variant that can be expressed in a cell model is disease- and mechanistically-specific for further ALS research.

In this proteogenomic workflow, we have designed an unbiased proteomics workflow to determine whether familial and sporadic mutations in *CCNF* implicated in ALS were found to activate pathways that were susceptible to neuronal cell death and cause aberrant E3 ligase activity of the SCF^(cyclin F)^ complex. Expression of selected ALS-linked *CCNF* mutations identified differences in the global proteome with many upregulated proteins found to cluster towards the Bad-Bax apoptosis pathway in transfected cells, while these same pathways were not activated in cells expressing the non-MND cyclin F mutation (R574Q) and *CCNF* wild-type control. Proteomics and biochemical analysis of induced pluripotent stem cells (IPSC) derived from patient fibroblasts bearing the ALS *CCNF*^*S621G*^ mutation showed similar activation profiles of the Bad-Bax apoptosis pathway. This was confirmed *in vivo* using zebrafish injected with ALS mutant *CCNF* mRNA, which increased activation of caspase-3 and caused aberrant branching of neurons in only disease-specific mutations, suggesting that ALS mutations in *CCNF* are highly specific in triggering neuronal cell death. Here, we thereby demonstrate a proof-of-concept proteogenomic workflow to screen new gene mutations (using *CCNF* as an example) to determine their potential to cause apoptosis and ALS pathogenesis using a combination of *in vitro* and *in vivo* models, proteomics, and biochemical assays. Our workflow aims to provide an efficient and timely means to prioritize gene candidates for further mechanistic studies, with potential application for the study of numerous disease-associated gene variants.

## Materials and Methods

### Cell Culture and Transfection

HEK293 cells were plated at 1 x 10^6^ cells in 100 mm plates and grown in high glucose DMEM supplemented with 10% heat inactivated FBS (F8192, Sigma Aldrich, MO, United States) in a 37°C heat-jacket humidified incubator with 5% CO_2_. After 48 hours of growth the cells were transfected with FLAG-tagged or mCherry-tagged *CCNF* cDNA constructs using Lipofectamine 2000 as previously described (Lee et al., 2017a, b).

Fibroblasts were obtained by skin biopsy from a 59-year-old symptomatic familial ALS patient (CCNF^*S621G*^) (Bax et al., 2019a), a 62-year-old pre-symptomatic familial ALS patient (CCNF^*S621G*^) and two healthy controls aged 57 (Balez et al., 2016) and 59 years old. All experimental protocols were approved by the University of Wollongong and Macquarie University Human Research Ethics Committees. The methods were carried out in accordance with the guidelines as set out in the National Statement on Ethical Conduct in Research Involving Humans, and informed consent was obtained from all donors. Fibroblasts were reprogrammed into induced pluripotent stem cells (iPSCs) and confirmed as pluripotent as we previously described (Bax et al., 2019a, b). The iPSCs were cultured on Matrigel (Corning) coated 6 cm tissue culture plates in TeSR-E8 (Stem Cell Technologies) at 37°C, 5% CO_2_ in a humidified incubator.

### Cell Lysis and Immunoprecipitation

HEK293 cells were harvested in ice-cold PBS and lysed using probe-sonication (10 s, setting 3, Branson Sonifier 450) in lysis buffer [50 mM Tris-HCl, 150 mM NaCl, pH 7.4, 1% (v/v) NP-40 (I8896, Sigma Aldrich)], containing 2 mM EDTA, 10 mM N-ethylmaleimide, complete protease inhibitor cocktail (4693159001), and phosSTOP (4906837001, Sigma Aldrich). iPSCs were harvested by gently rinsing in 1x PBS followed by manual scraping in RIPA buffer (50 mM Tris-HCl pH 7.4, 1% NP-40, 0.25% Na-deoxycholate, 150 mM NaCl, 1 mM EDTA, phosphate (PhosSTOP, Roche) and protease (cOmplete Protease Inhibitor Cocktail, Roche) inhibitors. Cellular debris was pelleted by centrifugation at 10,000 g for 10 minutes at 4°C. The cleared protein lysate was quantified by BCA (PIE23225, Life Technologies, CA, United States). Typically, 10 μg protein was used for immunoblotting and MS analysis. Proteins were denatured by boiling at 95°C for 5 minutes in 1x LDS buffer (NP0008) with 1x NuPAGE Reducing Agent (NP0009, Life Technologies).

For the E3 Ligase activity assay, Flag-M2 antibody (F1804, Sigma Aldrich) was used to immunoprecipitate cyclin F and associated proteins. 2 μg antibody was used per 500 μg cellular protein. A pre-immune control IgG (5415, Genesearch, QLD, Australia) immunoprecipitation was carried out in triplicate and used as a negative control. Protein A/G beads (88803, Life Technologies) were used to capture the antibody: protein complex. The beads were washed in lysis buffer and resuspended in E3LITE Assay buffer (100 mM Tris-HCl, pH 8, 10 mM MgCl_2_, 0.2 mM DTT).

### Immunoblot Analysis

Denatured proteins were separated by gel electrophoresis using 4–15% Tris-glycine gels (4565083, BioRad, CA, United States) according to manufacturer instructions. Separated proteins were transferred to nitrocellulose membrane using the Bio-Rad Turbo Transfer System using the “Mixed molecular weight” protocol (7 mins, 13 V, 1.3 A). Membranes were rinsed with deionized water and blocked in Odyssey Blocking buffer (92740003, LI-COR, NE, United States) diluted 1:1 in TBS for 1 hour at room temperature with shaking. Membranes were then incubated in either antibodies (against actin (A1978, Sigma Aldrich), total Bad (9239), phospho-Bad ser136 (4366) Caspase-3 (96625), Bcl-2 (sc1382), Bik (sc365625), Bax (sc7480), Bid (sc373939), diluted 1:1000 in 3% (w/v) BSA in TBS-T [50 mM Tris-HCl pH 7.4, 150 mM NaCl, 0.1% (v/v) Tween-20] overnight at 4°C with rocking. Membranes were then incubated in fluorescently labeled IRDye 800CW Goat Anti-Rabbit IgG or IRDye 700CW Goat Anti-mouse IgG secondary antibodies (LI-COR). Proteins were imaged using a LI-COR CL-X, and densitometry analysis was conducted using Image Studio Lite ver 5.2.

### E3 Ligase Activity Assay

The E3 ligase activity of the different cyclin F variants was assessed using E3LITE Customizable Ubiquitin Ligase Kit (E2 UBE2D3, Ubiquitin-Lys48 ubiquitin) (UC101, Life Sensors) and was carried out according to manufacturer’s instructions (96-well plate format) with slight modifications to the incubation time which was increased from 30 to 90 minutes. Luminescence was measured using ECL on a Pherastar plate reader. Immunoprecipitation (IP) efficiency was confirmed by immunoblot to confirm E3 ligase measurements were carried out on equal immunoprecipitated cyclin F amounts.

### Trypsin Digestion

Proteins were reduced and alkylated with 10 mM DTT and 55 mM iodoacetamide (IAA), respectively, and digested with trypsin (1:50 enzyme: protein) (V5111, Promega, WI, United States) overnight at 37°C. The digestion was inactivated by the addition of 2 μL of formic acid. Tryptic peptides were desalted on a pre-equilibrated C_18_ Sep-Pak cartridge (A57003100K, Agilent, CA, United States) and eluted in 50 % (v/v) ACN, 0.1% (v/v) formic acid, and dried under vacuum centrifugation.

### Reverse Phase C_18_ Liquid Chromatography-Mass Spectrometry (RP-LC-MS/MS)

Lyophilized peptides were resuspended in 0.1% FA and bath sonicated for 20 minutes. The resuspended peptides are then centrifuged at 14,000 g for 15 minutes to remove any insoluble debris, and the clarified peptides were analyzed by LC-MS/MS. The peptides were separated on an Ultimate 3000 nanoLC (Thermo Fisher Scientific) fitted with the Acclaim PepMap RSLC column particle size of 2 μm, diameter of 0.075 mm and length of 150 mm (Thermo Fisher Scientific), making use of a 60 min gradient (2–80% v/v acetonitrile, 0.1% v/v formic acid) running at a flow rate of 300 nl/min. Peptides eluted from the nano LC column were subsequently ionized into the Q Exactive Plus mass spectrometer (Thermo Fisher Scientific). The electrospray source was fitted with an emitter tip 10 μm (New Objective, Woburn, MA) and maintained at 1.6 kV electrospray voltage. The temperature of the capillary was set to 250°C. Precursor ions were selected for MS/MS fragmentation using a data-dependent “Top 10” method operating in FT acquisition mode with HCD fragmentation. FT-MS analysis on the Q Exactive Plus was carried out at 70,000 resolution and an AGC target of 1 x 10^6^ ions in full MS. MS/MS scans were carried out at 17,500 resolution with an AGC target of 2 x 10^4^ ions. Maximum injection times are set to 30 and 50 milliseconds, respectively. The charge exclusion was set to unassigned and 1+ with a dynamic exclusion of 20 seconds. The ion selection threshold for triggering MS/MS fragmentation was set to 25,000 counts, and an isolation width of 2.0 Da was used to perform HCD fragmentation with a normalized collision energy of 27.

Raw spectra files were processed using the Proteome Discoverer software 2.3 (Thermo) incorporating the Sequest search algorithm. Peptide identifications were determined using a 20-ppm precursor ion tolerance and a 0.1-Da MS/MS fragment ion tolerance for FT-MS and HCD fragmentation. Carbamidomethylation modification of cysteines was considered a static modification while oxidation of methionine, deamidation of asparagine and glutamine, GlyGly residues on lysine, and acetyl modification on N-terminal residues were set as variable modifications allowing for maximum two missed cleavages. The data was processed through Percolator for estimation of false discovery rates. Protein identifications were validated employing a q-value of 0.01. Label-free quantitation (LFQ) using intensity-based quantification was carried out. LFQ was carried out according to default parameter settings in the Proteome Discoverer 2.3 Software (Thermo). Briefly, peptide spectral matches (PSM) were filtered using a maximum delta Cn of 0.05, rank of 0, and delta mass of 0 ppm. PSMs and peptides were, respectively, validated using a strict FDR for PSMs of 0.01 and 0.05 for a relaxed FDR. Peptides shorter than 6 amino acids were filtered out. PSMs were chromatographically aligned for each input file in a sample set with a mass tolerance of 10 ppm and a maximum retention time (RT) shift of 10 minutes. Peptide groups used for protein quantification were analyzed using the default parameters which set a peptide as unique if it is included in only one protein group. The quantification was processed using unique and razor peptides (peptides shared among multiple proteins group or proteins) with the precursor abundance based on the intensity. Protein abundance was calculated as a sum of the individual peptide group abundances and the ratio was based on pairwise ratio using a geometric median of the peptide group ratios. An ANOVA test was used for the hypothesis test and uses the background population of ratios for all peptides and proteins to determine whether any given single peptide or protein is significantly changing relative to that background (Proteome Discoverer User Guide Software Version 2.3, Thermo).

Biological pathway analysis was carried out using PANTHER GO^1^ (Mi et al., 2019) and predicted pathway and biological process activation was carried out using Ingenuity Pathway Analysis (QIAGEN, Version June 2018).

### Generation of Zebrafish Overexpressing Cyclin F

Zebrafish were bred and maintained under established conditions (Westerfield, 2007) and all husbandry and experimental procedures were performed in compliance with the Animal Ethics Committee and Biosafety Committee, Macquarie University (AEC Reference No. 2015/034; 2017/019; NLRD 5201401007). Transgenic zebrafish that express blue fluorescent protein selectively in motor neurons on a TAB_WT background were used for this study [*Tg(-3mnx1:mTagBFP*)]^*mq10*^ (Don et al., 2017; Formella et al., 2018; Svahn et al., 2018). Only morphologically normal embryos were used for analysis, and all embryos were stage-matched through somite counts at 24 hpf.

The mCherry-*CCNF* plasmid (a gift from Michele Pagano, Addgene plasmid # 32975) (D’Angiolella et al., 2010) was used to generate constructs for this study. S621G, S195R and R574Q point mutations were introduced into *CCNF* within this plasmid using the Q5 Site-Directed Mutagenesis kit (NEB). Wild-type or mutant mCherry-tagged *CCNF* was then subcloned into the pCS2+ vector, and *CCNF* mRNA was generated from this vector using the mMESSAGE mMACHINE SP6 transcription kit (Thermo Fisher Scientific) as previously described (Hogan et al., 2017); 177 pg *CCNF* mRNA was injected into zebrafish embryos at the single-cell stage of development using a Picospritzer II (Parker Instrumentation) under a Nikon SMZ 745 stereomicroscope (Morsch et al., 2017). Embryos were screened for mCherry expression at approximately 6 hpf under a M165FC fluorescent stereomicroscope (Leica).

### Analysis of Axonal Branching in Zebrafish Embryos

Zebrafish embryos were manually dechorionated at 30 hours post-fertilization (hpf) and fixed in 4% PFA for one hour at room temperature. Fixed embryos were mounted in 3% methylcellulose and motor neurons expressing blue fluorescent protein imaged on a M165FC fluorescent stereomicroscope (Leica). The morphology of the ventral axonal projections of the first eight primary motor neurons immediately caudal to the yolk ball/tube boundary was assessed. Branching of the axon at or above the ventral edge of the notochord was considered aberrant (Lemmens et al., 2007). 41-46 embryos were assessed in each group across three experimental replicates.

### Caspase-3 Staining of Zebrafish Embryos

Embryos were manually dechorionated and euthanized at 24 hpf, then fixed in 4% PFA for 1 hour at room temperature. Immunostaining was performed using rabbit polyclonal cleaved caspase-3 antibody at 1:500 dilution (Cell Signaling Technology) and donkey anti-rabbit Alexa Fluor^®^ 488 secondary antibody at 1:500 dilution (Invitrogen) as previously described (Hogan et al., 2017). Embryos were mounted in 3% methylcellulose and imaged on a M165FC fluorescent stereomicroscope (Leica). Cleaved caspase-3 positive cells in the first five upper somites immediately caudal to the yolk ball/tube boundary were manually quantified. 30—40 embryos were assessed in each group across three experimental replicates.

### Acridine Orange (AO) Staining

Zebrafish embryos were manually dechorinated at 24 hpf, then placed in 5 μg/ml acridine orange solution (Sigma Aldrich) for 10 minutes. Embryos were rinsed, then mounted in 3% methylcellulose and imaged on a M165FC fluorescent stereomicroscope (Leica) scope. The number of acridine orange positive cells in the first 5 upper somites immediately caudal to the yolk ball/tube boundary manually quantified; 38–61 embryos were assessed in each group across three experimental replicates.

## Results and Discussion

With the emergence of whole genome sequencing that have facilitated the rapid discovery of new genes for basic scientific research and in medicine, more than 20 putative genes are now cited as causing ALS (Mejzini et al., 2019). These genes encode proteins that cover a diverse range of molecular functions, including free radical scavenging (superoxide dismutase SOD1), regulation of RNA homeostasis [TAR DNA-binding protein 43 (TDP-43) and fused in sarcoma (FUS)], and protein degradation through the ubiquitin-proteasome system (ubiquilin-2, cyclin F) and autophagy (sequestosome-1/p62, dynactin). It is well established that familial origins represent about 10% of all ALS cases, and while more than 20 genes are now identified as causing ALS, only few have been fully validated to determine the full extent of the perturbed molecular mechanisms and pathways. While sporadic ALS makes up ∼90% of patients, the origins remain unresolved but environmental factors and gene-environment interactions are thought to be intimately involved. A clear example of this can be seen in discordant identical twin (*n* = 3 pairs) and triplets (*n* = 1 set), where one twin has died of ALS, but the other remains healthy (Tarr et al., 2019). Combined differences were observed in longitudinal methylation (epigenetic), and transcriptomes implicated the genes *CCNF, DPP6, RAMP3*, and *CCS* in one set of twins, while longitudinal transcriptomics showed an enrichment of immune function genes and under-representation of transcription and protein modification genes in ALS. A possible explanation for disease discordance is that there is a complex interaction between genetic susceptibility and environmental factors (that may influence gene expression or protein modification and function) that ultimately drives disease pathogenesis.

Given the complexity in molecular origins of ALS, various molecular pathways have been implicated in ALS including disruption in DNA/RNA processing (Zhang et al., 2015, 2018), oxidative stress (Pollari et al., 2014), excitotoxicity (Foran and Trotti, 2009), protein misfolding and aggregation (McAlary et al., 2019), protein degradation (ubiquitin-proteasome and autophagy) (Shahheydari et al., 2017), ER stress (Walker and Atkin, 2011), and neuroinflammation (Liu and Wang, 2017). It is likely that many of these cellular processes are implicated at some point during disease, but what is less clear is how these (and other) molecular pathways interact with each other and converge together to cause ALS. It seems likely that the various initial triggers of disease (either genetic, environmental, or gene–environment interactions) must converge upon a common set of molecular pathways that underlie the pathogenesis of ALS. The ultimate identification of such convergent molecular mechanisms will be a key discovery towards understanding disease etiology and ultimately developing therapeutic targets for intervention. In this regard, it would be highly informative to use an unbiased screening approach to assess the activity of multiple pathways at the same time and investigate how these change over the course of disease and the interactions between pathways.

**FIGURE 1:**
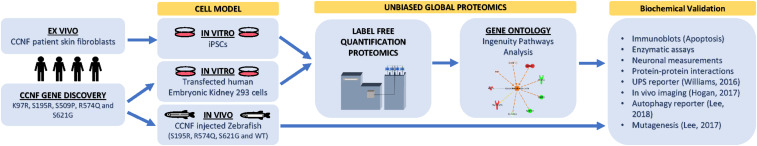
Proteogenomic workflow to determine whether potential ALS gene candidates cause disease. Discovery of gene mutations by whole-genome, whole-exome, or targeted sequencing. cDNA gene constructs candidate mutations are inserted into cell models (e.g., HEK293 or Neuro2A cells). Ex vivo models such as patient fibroblasts (if available) that contain the gene mutation can also be used or converted into iPSCs for further differentiation. Label-free quantitative proteomics is used to analyze and provide a profile of protein expression changes that cluster to biological processes and signalling pathways. These biological pathways can then be validated using standard biochemical methods and/or in animal models. Animal models, such as zebrafish, have the advantage of generating progeny relatively quickly and can be extended to study motor phenotypes.

We have developed a proof-of-concept integrated proteogenomics workflow (Figure 1) to provide broad insight into disease mechanisms through the identification of *CCNF* as a new ALS gene, and identification of disruption in multiple molecular pathways that converge to cause neuronal cell death (neurodegeneration). Pathways involved with activating apoptosis in cells expressing *CCNF* mutations were validated using various models, including cell lines, patient-derived iPSCs, and transgenic zebrafish. This integrated study demonstrates the utility and workflow for applying unbiased proteomics to ALS gene discoveries to determine whether new genetic variants are indeed potentially disease-causing mutations.

### Unbiased Proteomics to Identify Perturbed Pathways From Disease-Specific Mutations

Mutations in the *CCNF* gene have been established as a cause of ALS and FTD in a large Australian family, and expression of mutant cyclin F proteins in both cell line and zebrafish models have been shown to cause dysregulation of the UPS system (Williams et al., 2016; Hogan et al., 2017; Lee et al., 2017a, b). In previous studies, we have shown that expression of mutant cyclin F^*S621G*^ causes elevated Lys48 ubiquitylation of proteins and increased autophagy in human cell lines (Lee et al., 2017a, b). Additionally, injection of *CCNF*^*S621G*^ mRNA into zebrafish caused motor neuron axonopathies (Hogan et al., 2017). We aimed to explore whether this proteogenomics workflow would be useful for establishing whether a gene mutation(s) was indeed disease-causing, by using *CCNF* mutations previously reported by Williams et al. (2016) as a test case.

**FIGURE 2:**
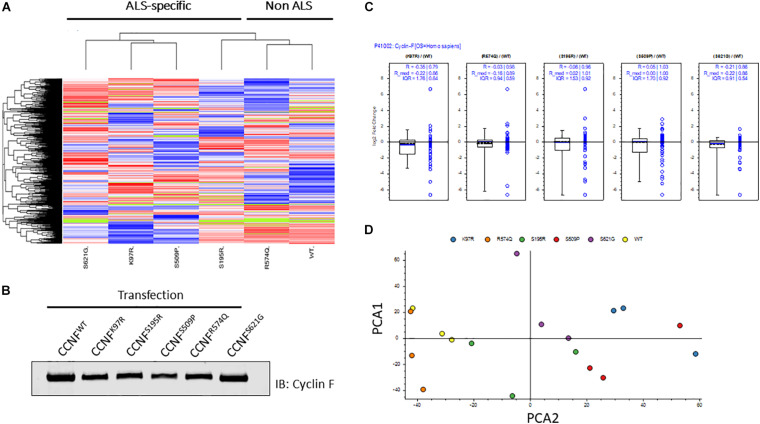
Label-free quantitative proteomics of HEK293 cells transfected with different cyclin F mutations (wild type, K97R, S195R, S509P, R574Q, S621G). **(A)** Hierarchical group clustering of proteome profiles from transfected cells showed ALS-specific and non-ALS mutations grouped together. Immunoblot analysis **(B)** and LFQ proteomics **(C)** of relatively equal cyclin F expression levels in transiently transfected HEK293 cells. **(D)** Two-dimensional principle component analysis (PCA) reflects the data quality and similarities of the proteome profiles between each biological replicate that are grouped by cyclin F mutations. Figures **(A,C,D)** were generated using Proteome Discoverer 2.3 (Thermo).

In this study, we selected familial ALS mutations in cyclin F (K97R, S195R, S509P, S621G) and one non-ALS public database SNP (R574Q) for analysis in our unbiased proteogenomic workflow. Expression of familial and ALS-specific (K97R, S195R, S509P, S621G) and non-ALS *CCNF* mutations (wild type and R574Q) were carried out in HEK293 cells, lysed and tryptically digested, and analyzed by LC-MS/MS. In total, we identified 5231 proteins with approximately 91% found in common between all samples indicating a high level of consistency from each proteome dataset. Using label-free quantitative proteomics to measure the relative abundance of each protein, we observed differential hierarchical clustering of the proteome datasets where both cyclin F wild type and R574Q displayed similar proteomic profiles across all identified and differentially expressed proteins, while the proteomic profiles of cells expressing cyclin F S621G, K97R and S509P appeared to cluster together (Figure 2A). This was intriguing as the proteome profile of cells expressing only the ALS-specific mutations clustered together. To ensure that these observations were not due technical variations, we examined the expression levels of cyclin F by immunoblot analysis (Figure 2B) and as measured by mass spectrometry (Figure 2C) and confirmed that the proteome similarities between ALS and non-ALS mutations were not due to the effects of transient transfections and differences in cyclin F protein expression. We next carried out an additional data quality control by principal component analysis (PCA) to examine whether there were variations between the individual biological replicates analyzed that may explain how the proteomes of ALS-specific mutations in cyclin F clustered together. Two-dimensional PCA analysis showed a high level of consistency amongst the samples analyzed, with biological replicates grouped together by cyclin F mutation (Figure 2D). Therefore, the data quality provided us with confidence that the proteomic profiles (and the lists of differentially expressed proteins) were due specifically to the expression of cyclin F mutations, which enabled us to extend the analysis to focus on perturbed cellular pathways.

**FIGURE 3:**
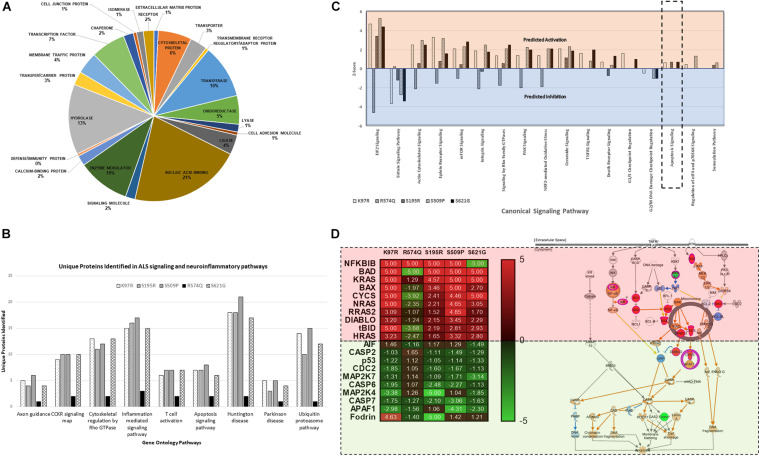
**(A)** Gene ontology (GO) distribution of the global proteome of HEK293 cells expressing cyclin F variants under the “Protein Class” category demonstrating proteins are classified as having molecular functions: nucleic acid binding, hydrolase, and transferase activity. **(B)** Selected gene ontology (GO) annotations of unique proteins clustered to selected pathways in neuroinflammation and ALS signalling. **(C)** Ingenuity pathway analysis (IPA) of the proteomic profile of cyclin F variants predicts activation and inhibition of various canonical cellular pathways. Apoptosis signaling was predicted to be activated in cells expressing cyclin F K97R, S195R, and S621G. Orange and blue annotation refer, respectively, to predicted activation and inhibition as determined by IPA. **(D)** Heatmap of label-free proteomics of differentially expressed proteins in the apoptosis signaling pathway and IPA prediction of downstream effects (log2 values shown; values shown as 5 and −5, respectively, indicate detection of the presence and absence of a protein and are not measured values). Red and green annotations refer, respectively, to experimentally measured values. Orange and blue annotations and lines refer, respectively, to predicted activation and inhibition as determined by IPA. Filled and dashed lines, respectively, refer to direct and indirect causal relationships.

Proteome profiles for HEK293 cells expressing cyclin F gene variants were analyzed by Gene Ontology using PANTHER GO^2^ and observed the distribution of proteins within “Protein Class” categories with nucleic acid binding (21%), hydrolase (13%), and transferase (10%) making up the top three functional categories for proteins identified (Figure 3A). The distribution of the different protein classes was similar and consistent across all proteomic profiles, and no differences were distinguished between proteins from each *CCNF* variant. Therefore, we carried out an analysis of protein identifications found uniquely in each of the *CCNF* variants (compared to the WT) (Figure 3B) and filtered the signaling pathways to those relevant to ALS pathogenesis and neuroinflammation. Interestingly, we observed unique proteins in cells expressing the *CCNF* ALS gene mutations but not in the wild type that are classified in the GO categories: ubiquitin-proteasome (such as UBE2E2 and WWP2), inflammation (such as STAT6 and CAMK2B), Huntington’s (such as TNFAIP8 and OPTN), Parkinson’s (such as MAPK7 and NDUFV2) and apoptosis signaling (such as JUN and MAP4K5). While these unique proteins identified in cyclin F variants gave us some clues to the pathways affected, we analyzed the proteomics data with their expressional changes using ingenuity pathway analysis^3^ (IPA) to calculate activation Z-scores using individual protein expression values and grouped according to their canonical cellular pathways. The predicted activation or inhibition scores enabled us to interrogate our dataset further and to provide confidence to direct our validation studies.

We analyzed the proteomic profiles of the different cyclin F variants using each protein’s abundance ratio (relative to the wild type control) as calculated by label-free proteomics. Interestingly, IPA determined that various canonical pathways were similarly predicted to be activated (such as actin cytoskeleton signaling) or inhibited (such as sirtuin signaling) in cells expressing the cyclin F variants with K97R, S195R, S509P and S621G mutations (Figure 3C). These four mutations were identified in various patients with ALS and FTD (Williams et al., 2016). In contrast, the cyclin F variant R574Q had a similar pathway profile to the wild type control and was characterized to be a database SNP and not found in ALS and FTD patient cohorts. This database SNP also ensured that an appropriate negative control was included in this proteomic study that is not known to cause ALS and/or FTD. Given the various reported cellular pathways that are perturbed at the onset and during ALS and FTD pathogenesis, our goal was to determine (i) whether mutations identified by GWAS studies were disease-causing and (ii) to focus on the end-point of disease which is fundamentally characterized by neuronal cell death (apoptosis).

Ingenuity pathway analysis predicted activation of the apoptosis signaling pathway in cells expressing cyclin F K97R, S195R, and S621G and therefore we focused on the identified proteins within the apoptosis pathway that were differentially expressed or perturbed (Figure 3D). Using the label-free proteomics data and IPA, we observed that the intrinsic pathway of apoptosis was predicted to be activated with various key pro-apoptosis players such as the Bcl-2 family including the pro-apoptotic proteins Bcl-2 associated agonist of cell death (Bad), Bcl-2 homologous antagonist killer (Bax), and BH3-interacting domain death agonist (Bid), which were all differentially upregulated in disease-causing cyclin F variants. The activation of the apoptosis pathway in K97R, S195R and S621G was not due to the transient transfection procedure since both cyclin F wild-type and the R574Q variants were also transfected using the same procedure without activating the apoptosis pathway. Since these pathways are based on IPA predictions using experimentally obtained label-free proteomics data, we validated various components of the apoptosis pathway by immunoblot analysis.

### Biochemical Validation of the Bad-Bax Apoptosis Pathway in Cells Expressing Cyclin F Variants

The Bcl-2 family consists of approximately 25 proteins that are involved with promoting or inhibiting apoptosis by controlling mitochondrial outer membrane permeabilization and release of cytochrome c (Kale et al., 2018). Bad/Bax/Bak are believed to initiate apoptosis by forming a pore in the mitochondrial outer membrane that allows cytochrome c to escape into the cytoplasm and activate the pro-apoptotic caspase cascade (Kalkavan and Green, 2018). While the anti-apoptotic Bcl-2 and Bcl-xL proteins promote cell survival by binding to Bad and Bak and inhibiting cytochrome c release through the mitochondrial pore and preventing the activation of the cytoplasmic caspase cascade (Shamas-Din et al., 2013). Bad mediates cell survival by heterodimerizing two anti-apoptotic proteins: Bcl-xL and Bcl-2. Following the activation of the Bad’s targeted proteins is the oligomerization of Bax and Bak monomers by Bim and truncated tBid to mediate the mitochondrial outer membrane permeability. Cytochrome c is released from the mitochondria to promote caspase-9 activation, which leads to the cleavage of caspase-3, caspase-7 and poly(ADP-ribose) polymerase (PARP), activating cellular apoptosis.

**FIGURE 4:**
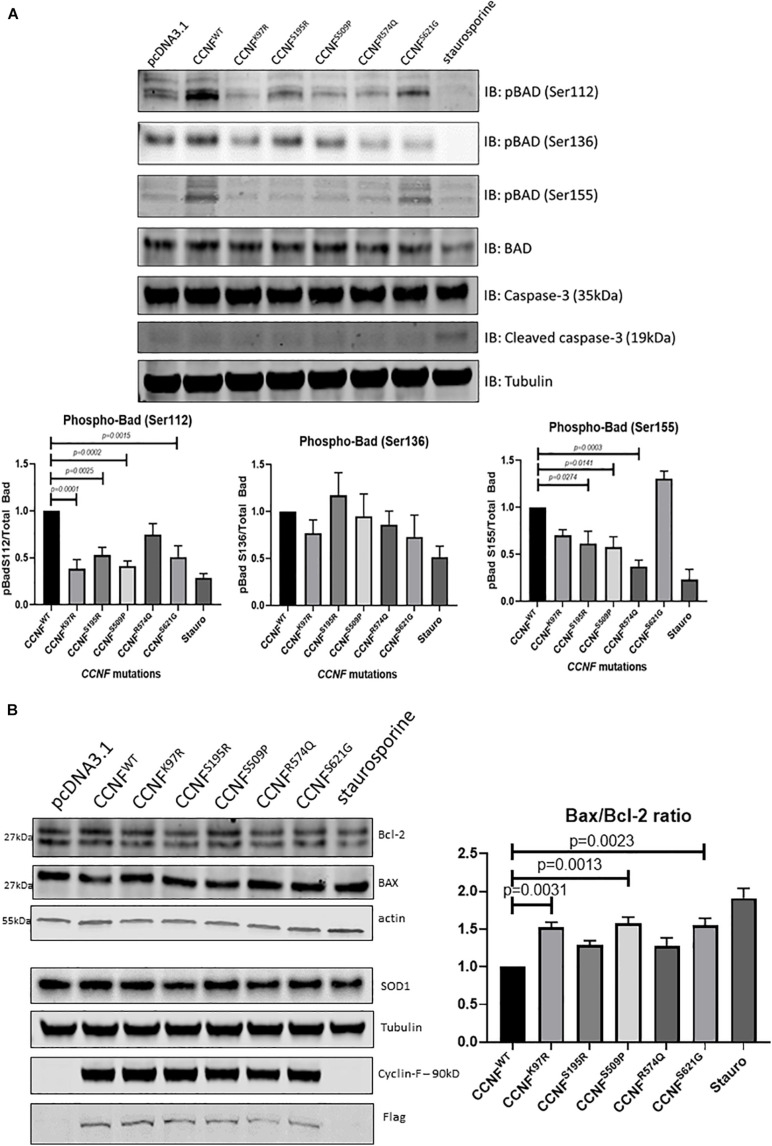
**(A)** Phosphorylation of Bcl-2 associated agonist of cell death (Bad) at Ser112, Ser136, and Ser155 reveals activation of the intrinsic apoptosis pathway. pBad (Ser112) was decreased in the cyclin F variants K97R (0.385-fold, *p* = 0.0001, *n* = 4), S195R (0.529-fold), S509P (0.413-fold), and S621G (0.508-fold) when compared to the cyclin F wild-type control (one-way ANOVA with Dunnett’s multiple comparison test, *n* = 4). pBad (Ser155) was decreased in only S195R (0.616-fold), S509 (0.578-fold), and R574Q (0.37-fold) when compared to the cyclin F wild-type control (one-way ANOVA with Dunnett’s multiple comparison test, *n* = 4). **(B)** Immunoblot analysis of Bcl-2, Bax, and SOD1 from cells expressing cyclin F wild-type and variants (K97R, S195R, S509P, R574Q, S621G) reveals increased Bax/Bcl-2 ratios in K97R (1.53-fold), S509P (1.58-fold), and S621G (1.55-fold) (one-way ANOVA with Dunnet’s multiple comparison test, *n* = 3) suggesting increased stimulation of apoptosis.

Moreover, the pro-apoptotic activity of Bad is regulated through its phosphorylation and sequestration to the cytosol by 14-3-3. Thus, one of the main markers to determine the level of apoptosis activation through this pathway is to measure the phosphorylation status of Bad. Seven phosphorylation sites have been identified on Bad: Ser112, Ser128, Ser136, Ser155, Ser170, Thr117 and Thr201. These sites are phosphorylated and regulated by a variety of kinases (Klumpp and Krieglstein, 2002; Burlacu, 2003). For example, dephosphorylated or reduced phosphorylation levels of Bad at Ser112, Ser136, and/or Ser155 (which are regulated by Akt, p70S6 kinase, and PKA) allows Bad to form a heterodimer with Bcl-2 and Bcl-xL blocking the Bcl-2 pro-survival activity and thus allowing Bax/Bak-triggered apoptosis to occur. Immunoblot analysis of phospho-Bad at Ser112, 136 and Ser155 from cells expressing the cyclin F wild type and variants K97R, S195R, S509P, R574Q, and S621G revealed no statistically significant phosphorylation differences at Ser136. However, there were observable decreases in phosphorylation at Bad, pBad (Ser112) and pBad (Ser155) (Figure 4A).

Notably, pBad (Ser112) was decreased in the cyclin F variants K97R (0.385-fold, *p* = 0.0001, *n* = 4), S195R (0.529-fold, *p* = 0.0025, *n* = 4), S509P (0.413-fold, *p* = 0.0002, *n* = 4), and S621G (0.508-fold, *p* = 0.0015, *n* = 4) when compared to the cyclin F wild-type control (Figure 4A). In addition, pBad (Ser155) was decreased in only S195R (0.616-fold, *p* = 0.0274, *n* = 4), S509 (0.578-fold, *p* = 0.0141, *n* = 4) and surprisingly R574Q (0.37-fold, *p* = 0.0003, *n* = 4) when compared to the cyclin F wild-type control (Figure 4A). The levels of pBad at the different sites can be regulated at various stoichiometries as they are influenced by i) specific environmental cues and ii) various upstream kinase(s) since multiple kinases can converge to phosphorylate the same site. This makes it difficult to determine the upstream changes that have led to the activation of Bad and the apoptosis pathway. What is apparent in this study, however, is that the decreased pBad levels at Ser112 and Ser155 would suggest that cells expressing cyclin F disease variants have increased apoptosis activity which supports the IPA predictions of the global proteomics analysis (Figure 3D).

To determine whether other components of the Bad-Bax apoptosis pathway were also activated in cells expressing cyclin F variants, we immunoblotted the pro-apoptotic protein Bax and compared the ratio to the anti-apoptotic Bcl-2 (Bax/Bcl-2) (Figure 4B). We observed increased ratio of Bax/Bcl-2 (pro-apoptosis/anti-apoptosis) in cyclin F variants K97R (1.53-fold, *p* = 0.0031, *n* = 3), S509P (1.58-fold, *p* = 0.0013, *n* = 3) and S621G (1.55-fold, *p* = 0.0023, *n* = 3) compared to the wild-type control. Superoxide dismutase is an enzyme responsible for reducing free superoxide radicals and is implicated in apoptosis and familial ALS. In this study, SOD1 and SOD2 were upregulated in only cyclin F disease-causing variants; however, immunoblot validations did not detect any observable differences in SOD1 abundance between the cyclin F variants. Importantly, we did not observe any differences in apoptosis in cells expressing the non-MND cyclin F mutation (R574Q). Taken together, immunoblot validation of the Bad-Bax apoptosis pathway (i.e., phosphorylation of Bad and Bax/Bcl-2 ratios) confirmed that cells expressing the cyclin F ALS variants activated apoptosis, giving us confidence that our proteogenomic workflow was capable of determining whether potential gene mutations are indeed disease-causing.

### Induced Pluripotent Stem Cells From ALS Patients Harboring the Cyclin F S621G Mutation Activate the Apoptosis Pathway

**FIGURE 5:**
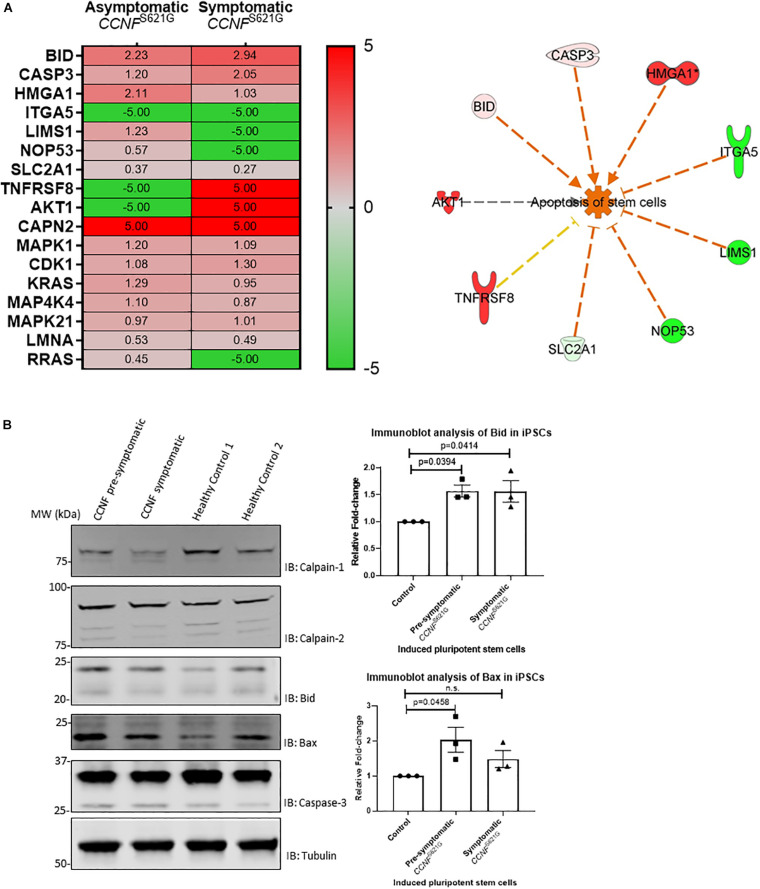
**(A)** iPSCs from asymptomatic and symptomatic ALS patients harboring the *CCNF* S621G mutation were subjected to global proteomics analysis. IPA of the quantitative proteomics data predicted activation of the apoptosis pathway. Red and green annotations refer, respectively, to experimentally measured values. Orange and blue annotations and lines refer, respectively, to predicted activation and inhibition as determined by IPA. Dashed lines indicate an indirect causal relationship (log2 values shown; values shown as 5 and −5, respectively, indicate detection of the presence and absence of a protein and are not measured values). **(B)** Immunoblot analysis of iPSCs from asymptomatic and symptomatic ALS patients harboring the *CCNF* S621G mutation compared to healthy controls reveals increased expression of pro-apoptotic proteins Bid and Bax. One-way ANOVA with Dunnett’s multiple comparison test (*n* = 3).

Given the limitations of overexpressing a transgene in a non-neuronal cell line (HEK293) for ALS research, we next tested iPSCs from a symptomatic ALS patient with the cyclin F S621G mutation, an asymptomatic or pre-symptomatic family member with the same mutation, and two healthy controls. The advantage of using iPSCs for this proteogenomic study is i) the uniform expression of the cyclin F S621G at endogenous levels and ii) the increased amount of material that can be obtained from these cultures that are sufficient for biochemical and proteomic analysis. We carried out proteomics analysis of the iPSCs from both the pre-symptomatic and symptomatic individuals. We detected differentially expressed proteins (such as Bid, caspase-3, and calpain) that are responsible for various stages of apoptosis signaling (Figure 5A) – the same pathway that was activated in the HEK293 cell lines expressing cyclin F variants. While the activation of apoptosis was anticipated in iPSCs (Activation *Z*-score = 1.67, *p*-value = 0.000396) derived from the symptomatic patient, it was also similarly predicted from iPSCs derived from the pre-symptomatic patient.

We carried out immunoblot analysis of calpain-1, calpain-2, Bid, Bax and caspase-3 to validate the proteomics results (Figure 5B). Calpain-1, calpain-2, and caspase-3 were detected, and their expression was determined to be similar (i.e., no statistical difference) between iPSCs derived from asymptomatic and symptomatic cyclin F S621G patients and healthy controls. Interestingly, we observed increased expression of the pro-apoptotic protein Bid and increased ratio of Bid/Bcl-2 in iPSCs derived from the symptomatic (Bid/Bcl-2 ratio 1.9-fold, *p* = 0.0255, *n* = 3) cyclin F S621G patient when compared to the healthy controls. There were no apparent differences in the Bid/Bcl-2 ratio between the pre-symptomatic patient and healthy controls. Additionally, increased expression of the pro-apoptotic protein Bax and increased ratio of Bax/Bcl-2 was also observed in iPSCs derived from pre-symptomatic (Bax/Bcl-2 ratio 1.7-fold, *p* = 0.0237, *n* = 3) and symptomatic (Bax/Bcl-2 ratio 1.87-fold, *p* = 0.0091, *n* = 3) cyclin F S621G patients when compared to the healthy controls (Figure 5B). The immunoblot analyses validate much of the proteomics data and the bioinformatics analyses with respect to predicting the activation of apoptosis from iPSCs from ALS patients expressing the cyclin F S621G mutation. This validation step also demonstrates a high level of confidence that the screening of HEK293 cell lines expressing cyclin F variants was indeed highly accurate in proteomic profiling to determine the activation status of apoptosis signaling and potentially other affected cellular pathways. The expression of transgenes in a cell- and/or species-specific model can be highly effective for determining how ’pathogenic’ a gene is as well as identifying the cellular pathways that may be perturbed and driving the biological processes towards cellular death.

### Cyclin F Mutations Cause Aberrant Lys48-Ubiquitin E3 Ligase Ubiquitylation Activity

**FIGURE 6:**
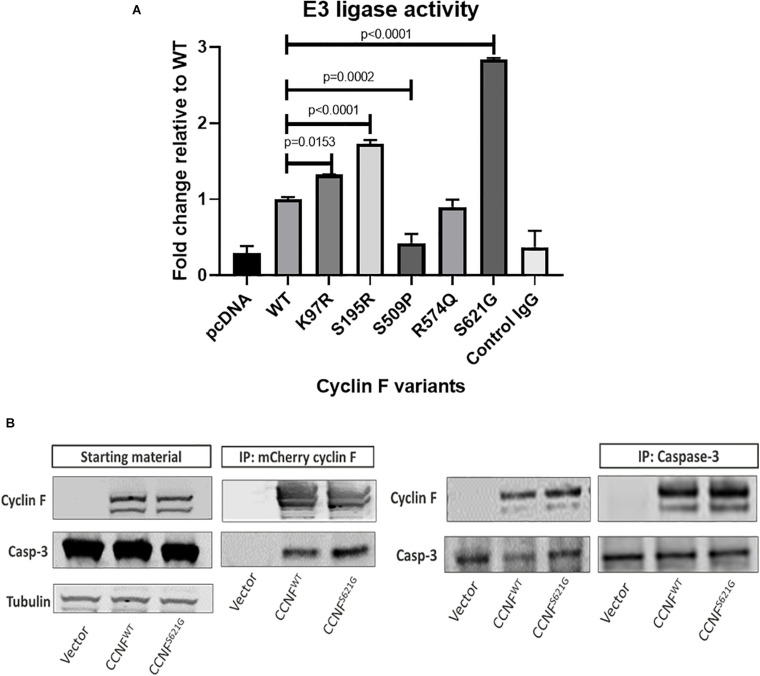
**(A)** Lys48-ubiquitylation E3 ligase activity assay of immunoprecipitated cyclin F from cells expressing cyclin F wild-type, K97R, S195R, S509P, R574Q, and S621G. **(B)** Cyclin F interacts with caspase 3. HEK293 cells transfected with mCherry-cyclin F and immunoprecipitated (left blot) shows interaction with endogenous caspase-3. The reverse immunoprecipitation to enrich caspase-3 showed the same interaction with cyclin F (right blot).

To gain insights into the mechanistic changes of these mutations to cyclin F activity, *in vitro* E3 ligase activity assays were carried out. Cyclin F is an F-box protein that is a component of the SCF^(cyclin F)^ complex, which is an E3 ligase responsible for ubiquitylating substrates for proteasomal degradation. To measure the enzymatic activity of cyclin F, we employed the E3LITE *in vitro* ubiquitylation assay with Lys48-ubiquitin using immunoprecipitated FLAG-cyclin F from biological replicates (*n* = 3) as previously described (Lee et al., 2017a, b) (Figure 6A). We observed increased E3 ligase activity from cyclin F variants K97R (1.324-fold, *p* = 0.0153, *n* = 3), S195R (1.73-fold, *p* ≤ 0.0001, *n* = 3) and S621G (2.83-fold, *p* ≤ 0.0001, *n* = 3) compared to the wild-type control. We have previously reported that the S621G mutation in cyclin F increases its E3 ligase activity by approximately 30–40% (measured for 30 minutes) compared to the wild type. We detected similar increases in ubiquitylation activity with the K97R and S195R mutations suggesting a gain-of-function in these cyclin F variants. Interestingly, the cyclin F variant S509P had dramatically reduced E3 ligase activity by 0.42-fold (*p* = 0.0002, *n* = 3) and suggests a loss-of-function to cyclin F. While the cyclin F variants examined in this study (except R574Q) activated apoptotic pathways that lead to neuronal loss [as determined by proteomics, immunoblot validations and patient information (Williams et al., 2016)], it suggests that both gain- and loss-of-function mutations in cyclin F are associated with the perturbation of downstream cellular pathways and cause proteostasis dysfunction. Therefore, the fine-tuning and regulation of cyclin F’s E3 ligase activity is highly important in maintaining cellular homeostasis.

### Cyclin F Interacts With Caspase-3

Given that caspase-3 can be regulated by ubiquitylation (Arama et al., 2007; Parrish et al., 2013) and is typically ubiquitylated for degradation by the proteasome (Choi et al., 2009), we hypothesized that cyclin F could potentially interact and/or ubiquitylate caspase-3. Caspase-3 contains two R-X-L motifs (residue 79–81 and 149–151) in its sequence, and cyclin F typically interacts with its substrates via this region (D’Angiolella et al., 2010). HEK293 cells expressing mCherry only, mCherry-cyclin F^*WT*^ and mCherry-cyclin F^*S621G*^ were immunoprecipitated using an RFP-Trap and immunoblotted for caspase 3 (Figure 6B). Immunoblot analysis revealed that caspase-3 co-immunoprecipitated with cyclin F. We performed the reverse immunoprecipitations of endogenous caspase 3 and immunoblotted for cyclin F, which revealed a direct interaction between caspase 3 and cyclin F.

### Cyclin F S195R and S621G Cause Increased Caspase-3 Activity and Aberrant Neuron Branching in Zebrafish

**FIGURE 7:**
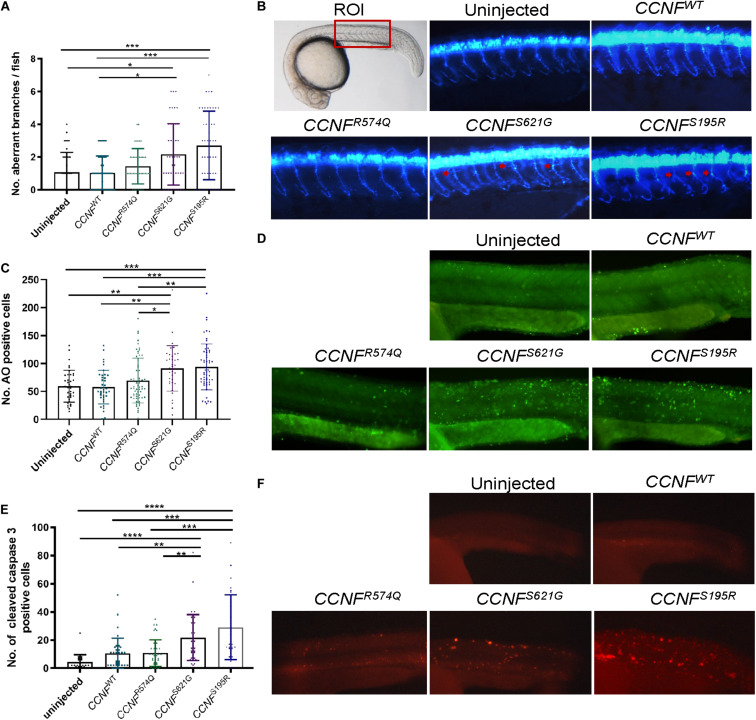
Measurements of aberrant neuron branching and apoptosis activation in zebrafish injected with *CCNF* gene variants S621G, S195R, R574Q, and wild type. **(A)** Blinded analysis of the number of aberrant branches in zebrafish expressing cyclin F S621G and S195R, respectively, had 2.01-fold (*p*-value = 0.006, *n* = 42) and 2.4-fold (*p*-value = 0.0011, *n* = 46) more aberrantly branched motor neurons compared to the wild type (one-way ANOVA). Region of interest (ROI) in zebrafish used for imaging. **(B)** Representative images of zebrafish expressing blue fluorescent protein in motor neurons used to analyse primary motor axon branching. Examples of aberrant branching indicated by red arrows. **(C)** Cyclin F S621G and S195R had displayed increased cleaved caspase-3 activity by 2.08-fold (*n* = 39, *p*-value = 0.0029 and 2.79-fold (*n* = 28, *p*-value = 0.0004) (one-way ANOVA adjusted with Kruskal–Wallis test). (**D**) Representative images of live zebrafish staining with AO. **(E)** AO positive cells in S621G and S195R were 1.4-fold (*p*-value = 0.0496, *n* = 37) and 1.45-fold (*p*-value = 0.0115, *n* = 57) higher, respectively, compared to the wild type (one-way ANOVA). (**F**) Representative images of zebrafish immunostained with cleaved caspase-3 primary antibody. *p ≤ 0.05, **p ≤ 0.01, ***p ≤ 0.001, ****p ≤ 0.0001.

To determine whether apoptosis pathway activation could be recapitulated *in vivo*, we injected zebrafish with *CCNF* mRNA as previously described (Hogan et al., 2017). The mRNA of *CCNF* variants S621G, S195R, and R574Q and wild-type *CCNF* were injected into zebrafish embryos at the single-cell stage of development, and various blinded measurements were made that included (i) the aberrant number branches per fish, (ii) the number of cells positively stained for cleaved caspase-3, and (iii) the number of cells stained with acridine orange as a measure of apoptotic cells (Figure 7). A blinded-analysis was carried out and determined that zebrafish expressing cyclin F S621G and S195R, respectively, had 2.01-fold (*p*-value = 0.006, *n* = 42) and 2.4-fold (*p*-value = 0.0011, *n* = 46) more aberrantly branched motor neurons compared to the wild-type and uninjected controls. The incidence of aberrant axonal branching in zebrafish expressing the non-ALS cyclin F variant R574Q did not change significantly (1.27-fold) compared to fish expressing wild-type cyclin F (Figure 7A). We then carried out immunohistochemistry analysis of cleaved caspase-3 by counting the number of positive cells in injected zebrafish, as a crude measure of the level of enzymatic activity of caspase-3. Similarly, both cyclin F S621G and S195R injected zebrafish had increased cleaved caspase-3 activity by 2.08-fold (*p*-value = 0.0029, *n* = 39) and 2.79-fold (*p*-value = 0.0004, *n* = 28), respectively, when compared to the cyclin F wild-type (Figure 7B). The cyclin F R574Q (1.03-fold) did not show any significant differences compared to the wild type. Lastly, we carried out acridine orange (AO) staining to determine the number of apoptotic cells in zebrafish injected with *CCNF* variants. Consistent with the previous results, the number of AO positive cells in S621G and S195R were 1.4-fold (*p*-value = 0.0496, *n* = 37) and 1.45-fold (*p*-value = 0.0115, *n* = 57) higher, respectively, compared to the wild type. The number of AO stained cells in zebrafish injected with R574Q (1.07-fold) and the wild type was not significantly different (Figure 7C). Collectively, these three *in vivo* experiments to measure (i) aberrant neuron branching, (ii) level of cleaved caspase-3, and (iii) the amount of apoptotic cells provide further validation that increased activation of apoptosis occurs in zebrafish injected with ALS-specific *CCNF* gene variants.

### Proteogenomics Workflow Reveals Potential Disease-Causing Mutations

The pathological hallmark in almost all cases of ALS is the presence of aggregated, acetylated (Cohen et al., 2015), ubiquitylated (Neumann et al., 2006), and phosphorylated (Hasegawa et al., 2008) TDP-43 in post-mortem patient brain tissue (Neumann et al., 2007). Numerous mechanisms have been implicated in the pathogenesis of ALS which are related to mutations and/or proteostasis dysfunction that impact on various cellular pathways including intracellular transport (Walker and Atkin, 2011), protein folding and degradation (Shahheydari et al., 2017), cellular stress (Ling et al., 2013), and RNA metabolism (Zhang et al., 2015, 2018). However, the initial trigger(s) that sets off a chain of biological events that lead to motor neuron loss (and eventually ALS) is still not known.

Genome-wide association studies has rapidly accelerated the identification of new genetic causes of ALS with more than 20 putative ALS-causing genes now cited. This series of discoveries have led to a better understanding of disease mechanisms that have helped draw connections between these gene discoveries (and their reported canonical functions) to the pathological features observed in ALS patients. Moreover, identification of gene variants has also enabled the generation of *in vitro* and *in vivo* models that recapitulate various features of the disease despite most models artificially over-expressing a disease-linked protein. While there are bioinformatic tools available to predict if a gene variant may be pathogenic, one major challenge is actually verifying these in a model system with a method to confirm that changes to pathways related to neuronal damage and/or loss have occurred or are predicted to occur.

Using our proteogenomics workflow with *CCNF* gene variants, as a proof-of-concept, we expressed cyclin F into a HEK293 cell line given its utility to be grown easily in culture, withstand the transient transfection procedure, and express the transgene-of-interest with consistent high copy numbers without compromising cell viability. Label-free quantitative proteomics was used to identify and quantitate the proteome which has various advantages mostly because it is a cheaper and more suitable approach for the main purpose of identifying the effect of transgene expression on perturbed cellular pathways. Other quantitative proteomics techniques such as SILAC and TMT (Hedl et al., 2019) are much more accurate in their measurements of protein expression; however, they can often be costly and have limited benefit when determining bioinformatic predictions on perturbed cell pathways compared to LFQ proteomics. Given that the underlying biology for ALS, much like other neurodegenerative diseases, involves neuronal cell death, our criteria for determining whether a gene variant was pathogenic was to make predictions using our experimental proteomics data and bioinformatic tools (IPA) to establish if apoptosis pathways were activated or predicted to be activated. Other bioinformatic tools such as Reactome, MetaCore, and PathVisio can also be used to visualize -omics data matched to pathways (Cirillo et al., 2017). It should be noted that in our study, other molecular pathways were also predicted to be affected; however, our emphasis was to screen for gene variants and evaluate their effects on apoptotic pathways as a key feature of ALS.

Our proteomic findings provide us with confidence and experimental options with which to validate our *CCNF* gene variants (i) in various models (iPSCs and zebrafish) and (ii) for mechanistic studies (immunoblot analysis, enzymatic activity assays, and protein–protein interaction). Other validation experiments, such as GFPu reporter (Yang et al., 2015), FloIT (Whiten et al., 2016), live-cell imaging (Farrawell et al., 2018), and toxicity assays (McAlary et al., 2016), may also be considered to support predictions made from the proteomics experiments. Here, we demonstrated by proteomics analysis of iPSCs derived from fibroblasts of *CCNF*^*S621G*^ patients that transfection of HEK293 cells was an appropriate model of apoptosis pathway activation as verified by immunoblot analysis of the Bad-Bax pathway. Measurements of aberrant neuron branching, cleaved caspase-3, and acridine orange staining for apoptotic cells in zebrafish reaffirmed the predicted activation status of apoptosis from the initial proteomics screening of transfected HEK293 cells.

To provide some mechanistic insights, we demonstrate that *CCNF* gene variants found in patients with ALS had impaired cyclin F activity with increased E3 ligase activity in mutations K97R, S195R and S621G (gain-of-function), while the S509P mutation showed reduced/loss of E3 ligase activity (loss-of-function). Our non-ALS SNP mutation R574Q did not display any aberrant activity when compared to the wild-type control. This suggests that mutations in *CCNF* dysregulate the E3 ligase activity of the cyclin F protein. To correlate the proteomics screening which predicted apoptosis activation together with impairments to cyclin F E3 ligase activity, we hypothesized that cyclin F may be involved in the ubiquitylation of a key component in the apoptosis pathway. We performed immunoprecipitations of cyclin F and caspase-3, given that it contained two R-X-L binding motifs and as one of the main players of apoptosis, and confirmed that these proteins interact endogenously. The interaction between these proteins may provide important insight into the pathogenesis of ALS and warrant further investigation.

Taken together, we have demonstrated the advantage of employing a proteogenomics workflow to screen new ALS gene mutations and determine their pathogenic potential. Our approach allows for rapid unbiased analysis which generates a wealth of biological information to enable new hypotheses and new disease models to be developed.

## Data Availability Statement

The mass spectrometry proteomics data and additional tables are available on the ProteomeXchange Consortium via the PRIDE (Perez-Riverol et al., 2019) partner repository with the dataset identifier PXD021793 and doi: 10.6019/PXD021793.

## Ethics Statement

The studies involving human participants were reviewed and approved by the University of Wollongong Human Ethics Committee (HE 13/272). The patients/participants provided their written informed consent to participate in this study. The animal study was reviewed and approved by the Animal Ethics Committee and Biosafety Committee, Macquarie University (AEC Reference Nos. 2015/034 and 2017/019; NLRD 5201401007).

## Author Contributions

All authors were responsible for drafting, writing, and editing the manuscript, and all authors read and approved the final manuscript. FC, AD, AH, SR, JD, MV, and AL were responsible for carrying out the cell line and proteomics experiments, and bioinformatics analyses. CS, SM, and LO were responsible for generating the IPSC cells. AH, MW, ED, and ASL were responsible for all zebrafish work. JY, TC, TH, AW, SY, MM, BS, IB, and RC provided intellectual input and design of some of the experiments.

## Conflict of Interest

The authors declare that the research was conducted in the absence of any commercial or financial relationships that could be construed as a potential conflict of interest.

## Footnotes

1 www.pantherdb.org

2 http://pantherdb.org

3 https://www.qiagenbioinformatics.com/products/ingenuity-pathway-analysis
